# ‘The Devil has entered you’: A qualitative study of Men Who Have Sex With Men (MSM) and the stigma and discrimination they experience from healthcare professionals and the general community in Bosnia and Herzegovina

**DOI:** 10.1371/journal.pone.0179101

**Published:** 2017-06-07

**Authors:** Stela Stojisavljevic, Bosiljka Djikanovic, Bojana Matejic

**Affiliations:** 1 Public Health Institute Republic of Srpska, Banja Luka, Bosnia and Herzegovina; 2 Institute of Social medicine, Faculty of Medicine, University of Belgrade, Serbia; 3 Centre–School of Public Health, Faculty of Medicine, University of Belgrade, Serbia; Masonic Cancer Center, UNITED STATES

## Abstract

Men who have sex with men (MSM) are often exposed to unequal treatment in societies worldwide as well as to various forms of stigma and discrimination in healthcare services. Bosnia and Herzegovina (B&H) is a postconflict developing country located in Southeast Europe and the Western Balkans, where little is known about the experiences of MSM regarding their communities and interactions with healthcare services. The aim of this study was to explore the types of experiences MSM face and to assess the level of stigma and discrimination they are exposed to in this setting. We conducted twelve in-depth face-to-face interviews with MSM who were 16 to 45 years old and residing in B&H. The main findings indicated that they all experienced various levels of stigma, discrimination, prejudice and inequities in treatment and attitudes from different segments of society, including the health care sector, that prevented them from fully developing their human and health potential. Additionally, these experiences were adversely related to opportunities to receive good quality health care services due to the insufficiently educated and old-fashioned health professionals who sometimes believed in black magic practices. The findings present numerous opportunities for educational trainings and structural reform to create a society that provides and guarantees equal opportunities for all.

## Introduction

Men who have sex with men (MSM) often experience social exclusion, marginalization, violence and different forms of stigma and discrimination [1–3]. These phenomena are more or less present and documented worldwide, regardless of the large differences in the social, political, cultural and legal environments in which these people live [1–5]. Although civil societies have significantly progressed towards a vision of equality for all people regardless of sexual orientation, stigma and violence towards MSM still occur, and homophobia remains tolerated and supported by different policies [4]. The latest data show that sexual relationships among same-sex adults are still considered criminal offenses in 75 countries worldwide and are punishable by fines or imprisonment. Furthermore, in eight countries in Africa and southwestern Asia, the justice system includes even a death penalty for homosexuality, although this punishment is implemented in ‘only’ five countries in practice (Mauritania, the Sudan, Iran, Saudi Arabia and Yemen) [5]. Countries on the border of the European Union (Southeast Europe and the Western Balkans) have much more developed justice systems than these countries, but the law enforcement and MSM experiences differ in practice. These systems are mostly conditioned by the country’s relationship with the European Union i.e., membership, where respecting human rights and sexual freedoms are an absolute imperative. Other than official reports from the United Nations agencies and similar organizations, we found limited scientific studies on MSM populations in Southeast Europe and the Western Balkans in a literature review. Countries from this region were included in a large pan-European internet-based study on MSM called the European MSM Internet Study (EMIS) that was conducted in 38 countries (including Bosnia and Herzegovina, B&H); however, a national report was not produced in B&H, because the population size was too small [6]. However, the EMIS study provided valuable data related to homophobia, stigma, discrimination and health. The results revealed that the internalized homonegativity among MSM in B&H was among the worst of all countries involved in the study [7]. As reported by Ross et al., this type of self-discrimination is strongly influenced by the overall legal climate related to the recognition and rights of the MSM population, as well as by the level of inequalities in a country [7]. Additionally, the same authors hypothesized and confirmed by empirical data that internalized homonegativity influences outness, perceived control over sexual risk-taking behavior, and consequently HIV testing [7]. According to a national report on the MSM population in B&H, only 28.4% had disclosed their sexual orientation to their families, and 9.5% to their general practitioner (GP), i.e., family doctors [8]. The health of the MSM population is endangered through risky behaviors performed in sexual relationships, including having a number of non-steady partners and unprotected sex, as well as abstaining from sexually transmitted infection (STI) and HIV testing [7]. Results from national bio-bihevioral study that was carried out in 2015 confirmed existence of MSM risky behaviors in B&H: just 20.8% MSM used condom during each sexual intercourse with steady partner and 38.9% used condom everytime that they had sexual intercourse with an occasional partner while only 2% used condom during oral sexual intercourses [8]. These risky behaviors are likely to correlate with the high levels of internalized homonegativity present among MSM in B&H [7]. Indeed, findings from a national report indicated that 35.3% of MSM have received HIV testing and know their HIV status; however, 38.2% have never talked about their HIV status with their partner, which might function as a proxy for unprotected sex [8]. The same study identified that the prevalence of HIV in MSM population is 1%, and the prevalence of Hepatitis C and Heapatitis B is 0.5% [8]. The risk of HIV acquisition among MSM in B&H is thus high, and the most recent data indicated that almost every third person who is HIV-positive in B&H (31.6%) acquired HIV through homosexual contact [9]. This percentage might be even larger because the homosexual ways of HIV transmission are reported only when individuals have trust in healthcare professionals and do not fear that this information will cause stigma and discrimination. A national report on the status of lesbian, gay, bisexual and transgendered (LGBT) persons identified that more than a quarter of openly LGBT persons in B&H have had a negative experience with health care professionals: 27% with a doctor of family medicine; 22% with a gynecologist, and 37% with a urologist [10]. Furthermore, the situation is not better when considering health professionals: a national study on HIV, stigma and discrimination among healthcare professionals showed that 78% had heard or witnessed discriminating behavior towards patients infected with HIV [11].

Homophobia, stigma and discrimination present great challenges for the MSM population and can manifest different forms, ranging from everyday obstacles in organizing their personal lives to the problems at a much higher level. They involve structural factors that further barriers to the integration of MSM, regardless of whether they originate from civil society organizations, church and religious associations or law enforcement agencies and governments. However, different societies respond differently to MSM, and it is of utmost importance to explore these responses in the context of a specific social, political and cultural framework.

B&H is a multicultural country located in Southeast Europe and the Western Balkans region. It consists of three political entities (the Federation of Bosnia and Herzegovina, Republic of Srpska and Brčko District). After an extremely turbulent period at the end of 20^th^ century, the disintegration of former Yugoslavia, the war (1992–1995) and the reconstruction and reorganization of the country, B&H entered the new century in a difficult period of economic transition. The healthcare system, as well as all social systems of the country, experienced terrible consequences of the war and transition period, and there were numerous efforts among the international community and healthcare cooperatives to strengthen and promote health care services in this post-conflict country [12].

In the complex and multicultural environment of B&H, little is known about the MSM population. There are an estimated 6900 (4300–9500) MSM in B&H, representing approximately 0.57% of the adult male population [13]. The demographic characteristics of the MSM population in B&H who participated in the EMIS study showed that more than half had a tertiary education (53.8%) and had been employed (56.7%), whereas only every fifth MSM (22.1%) at the time of the survey was in a relationship with a man. The EMIS data indicated that in terms of internalized homonegativity/homophobia (IH), MSM in B&H had the second highest IH score in the 38 European countries in the study [7]. However, whether these data are representative of the entire MSM population is debatable, as they were based on a sample recruited through the Internet.

The MSM population in B&H is a particularly vulnerable. Although there is a law that prohibits discrimination based on sexual orientation and gender identity, it is not implemented to the full extent in practice, and a system for registering cases of discrimination has still not been developed [14]. Since 2009 when the „Law on Prohibition of Discrimination in B&H”was adopted, the Ombudsman institute in Bosnia and Herzegovina registered 38 cases of discrimination based on sexual orientation [15]. At the beginning of the work conducted by Ombudsman in B&H, the largest number of received complaints was related to discrimination at the workplace (mobbing) or on the grounds of ethnicity or national and social background, whereas discrimination based on sexual orientation was reported less often; however, that number is rising every year [16]. A report on hate crimes in B&H during a period of 8 months in 2013 documented 18 cases of hate crimes, 5 cases of discrimination on the basis of identity and over 20 cases of hate speech on the grounds of sexual orientation and/or gender [17]. Discrimination of the LGBT population is most often reflected in hate speech, offensive content through graffiti, endangering the freedom of gathering and expression, threats and cases of individual violence. To our knowledge there have been no attempts to date to examine the depth and complexity of the stigma and discrimination faced by MSM in B&H nor the relationship and attitudes of healthcare providers towards them. Given the unfavorable foundation characterized by insufficient legal support and protection of the rights of the MSM population in B&H, the above-mentioned factors can be assumed to influence the likelihood that MSM will receive necessary and good quality healthcare services [4, 18].

The aim of this study was to dertermine the experiences of the MSM population in B&H regarding the relationships with and attitudes of the general community, with a particular emphasis on the healthcare service and its role.

## Method

### Study design and selection of participants

This qualitative study was conducted in the form of in-depth interviews with members of the MSM population from different parts of B&H. To reach potential participants for an interview, we contacted two civil society roof organizations that address MSM related issues in B&H (‘Action Against AIDS’ (AAA) and ‘Association XY’) and networked with other civil society organizations that manage LGBT related issues in B&H. AAA is a non-governmental organization (NGO) located in Banja Luka that works with populations at an increased risk of HIV infection. AAA provides different activities including HIV/STI-related counseling; psychological counseling for the most at-risk populations (MARPs); distribution of information and educational materials, condoms and lubricants; outreach work related to HIV prevention; peer education for MSM; and organization of events (gathering) for MSM [19]. Association XY (XY) is an NGO with its head office in Sarajevo and a branch office in Banja Luka. XY primarily works with youth, especially those from vulnerable and unreachable populations. XY promotes the sexual and reproductive rights of all people by providing information and educational materials, advocating for better legislation policies and raising public awareness on the importance of reproductive health [20]. An invitation to participate in the study was placed on the web pages of these organizations for two weeks, along with basic information concerning the study (the study aim and methods, individuals involved in the study and method of protecting information about the potential participants). When applying to participate in interviewes, potential participants first contacted a delegated representative of the above-mentioned organizations and provided initial consent for the participation in the study as well as contact information. The interviewer then contacted the respondents by phone to arrange the place and time of the interview. Initially, we aimed to interview between 8 and 12 MSM, as we estimated that would be sufficient for achieving data saturation [21].

The criteria for participation in the in-depth interviewes were self-identification as a member of the MSM population, age of at least 16 years old and provision of informed consent to be interviewed. In total, twelve MSM responded to the invitation to participate in the study. All of them met the participation criteria and gave informed consent to be interviewed. No remuneration was provided for their involvement in the study. Data saturation was achieved after the tenth interview, and new themes did not emerge in the last two interviewes.

### Ethical consent

The principles of the Declaration of Helsinki were followed during the preparation and implementation of the study, i.e., the study team ensured that the respondents’ dignity, identity and right to data confidentiality were protected in all phases of research. All members of the study team signed a statement on data confidentiality. Prior to study implementation, consent from the ethical board of the Institute for Public Health in the Republic of Srpska (decision number 500-1282-6/15, as of May 20^th^ 2015) and the Public Health Institute of the Federation of Bosnia and Herzegovina (decision number S-01-01-7-154-7/15 as of May 15 ^th^ 2015) was obtained. Ethical boards of the Institute for Public Health in the Republic of Srpska and the Public Health Institute of the Federation of Bosnia and Herzegovina were introduced with all aspects of the study (study protocol, interview guide, informed consent procedure, evidence lists, etc.) and they approved the lack of parent or guardian consent for interviewees the age of sixteen and above. Before the interview was conducted, the participants were asked for their consent to participate in the study, as well as their consent to be recorded, which they confirmed with their signature, initials or some other code. The informed consent procedure was in line with the Code of Ethics of research with and about the children in B&H [22], according to which parental consent for participants above the age of fifteen was not required.

### Preparing and conducting the interview

Before the in-depth interviews, a guide for the interviewer was developed ("S1 File") and defined in accordance with the aims of the study and with the best practices for conducting qualitative research [23,24]. The interview guide contained a framework of the themes of interest and corresponding open-ended questions, such as questions related to societal attitudes towards the participants, considering their sexual orientation, and their different experiences with their families, friends, healthcare professionals and the general community.

Interviewes were held in five different towns in B&H that were convenient to the respondents. They also selected a location for the interview that they were comfortable with, such as hotel rooms, at NGOs, and private dwellings. The interviewer in this study was a medical doctor by profession, who had received a number of educational trainings related to HIV/AIDS and MSM and was experienced in conducting interviews, working with MSM, and performing public health research (SS, the first author of this paper).

### Qualitative data analysis

The audio-recorded interviews were conducted in Bosnian languages (Serbian, Croatian and Bosnian) and transcribed verbatim. The interview transcripts were used as the basic unit for the qualitative content analysis, together with the observation notes [23,24]. These data were analyzed according to a directed qualitative analysis approach, which meant that the quidance for the initial codes was based on theory and relevant research findings [23].

The analysis team consisted of the inteviewer and two researchers with experience in the field of qualitative research (BM and BD). The interviewer and researchers were fluent in both Bosnian and English, thus ensuring the quality of the translation of the transcripts into English.

All transcripts were read first to obtain an overall understanding of the data obtained from the respondents. After this phase of obtaining initial insight into the text, the transcripts were read in detail and the researcher broadly noted potential issues and concepts using pencil and printed text, underlining parts of the text that were particularly indicative. Relevant words, phrases, sentences or paragraphs of the text were thus marked (*labeling*).

The next phase included listing of potential subjects and organizing them into coherent categories (*coding and indexing*). Certain important activities, relevant attitudes, processes, and specific behaviors or concepts were marked, i.e., everything that had been highlighted as relevant to the researcher during the initial and detailed reading of the transcripts.

During the previous phase, many different codes were generated, which were then organized into larger categories. The researcher iteratively reviewed the transcripts, critically observing the codes and trying to organize and label them into certain subjects i.e., categories, impartially as possible. The next step was related to estimating the importance of certain categories and evaluating mutual relationships. All three researchers independently performed this process and discussed and agreed on the coding scheme and interpretation presented here.

## Results

Twelve respondents, who were members of the MSM population in B&H and aged between 16 and 45 years, participated in this study. In-depth interviews were conducted in July and August 2015. Each of the interviews lasted approximately one hour. Two of the interviewees refused to give permission to be recorded, and the interviewer thus took notes in a way that did not interfere with the dialogue, completing the text with more details after the interview had been conducted and noting observations.

The socio-demographic characteristics of the respondents are provided in Table 1. The highest proportion of respondents (two-thirds) were 20 to 25 years old. Four respondents who had a job were employed in a civil society organizations (NGO sector).

**Table 1:** Basic socio-demographic characteristics of the respondents.

Characteristics of respondents		n	%
**Age**			
	Up to 20 years	2	16.6
	20 to 25 years	6	50.0
	26 to 30 years	2	16.0
	Over 30 years	2	16.6
**Education status**			
	Attending secondary school	2	16.6
	Finished secondary school	3	25.0
	Attending university	3	25.0
	Finished university	4	33.3
**Employment**			
	Employed	2	16.6
	Unemployed	6	50.0
	Student/University studnet	4	33.3
**Persons they live with**			
	Partner	2	16.6
	Parents/brothers or sisters/grandmother	7	58.3
	Alone	3	25.0

### Attitudes of the general community towards MSM

The respondents’ experiences concerning their status in the general community in B&H were very coherent. All respondents reported that they felt unequal and discriminated against compared with the position of heterosexual men. They thought that society as a whole was still not ready to completely accept the LGBT population. They described numerous prejudices and stereotypes that they experienced that made them hide their real nature (“*we are behind a curtain****”***- Z. 24) and avoid publicly disclosing their status. Namely, when mentioning the fact that most respondents were not publicly outed, they stated that the ubiquitous homophobia they felt in the community presented the greatest barrier. They felt and experienced discrimination in various aspects of everyday life (Box 1).

**Box 1. Discriminations felt and experienced by the MSM population in B&H**

- Attitudes of the community concerning the MSM population that are expressed through hate speech, physical threats and attacks
- The passive position of the state, institutions and the media – the laws aimed at preventing discrimination exist but they are not enforced
- Obstacles concerning the labor market
- Difficulties in organizing everyday life
- Inability to get married and obtain the rights of married couples
- Absolute non-acceptance from all religious communities

When talking in detail about the understanding and conceptations that the public had about the MSM population, the respondents used numerous expressions that they thought best reflect the general attitudes towards them:

‘(…) they don’t consider it to be normal… it’s something that’s not good… something wrong… a disease… a deviation… a psychiatric disorder… something that terrifies people… an epidemic… people can’t stand it, they don’t want it to become a part of our community, our society… something that should be hidden… it is not acceptable… a taboo… something that should be eradicated… degenerates… if you want to offend somebody you tell them they are gay… we have become a swear word like any other swear word… a label… the consequence of evil forces… The Devil has entered you… black magic…’

On several occasions the dominant narrative directed towards the MSM population escalated into hate speech and concrete acts of violence were reported by some respondents. In their statements they reported the use of abusive language and insults that they remembered from childhood. They also claimed that it was sometimes enough for individuals to identify a ‘feminine detail’ *(D*., *16)* and that this feature would become the reason for public humiliation and harassment, which was often witnessed by very young respondent. At the same time, the respondents had no confidence in the institutions (police, the state prosecutor’s office) that had the jurisdiction to prosecute the perpetrator if their integrity was threatened. Although anti-discrimination laws existed, our interviewees claimed on several occasions that these laws were not enforced in practice and that MSM were actually unprotected:

*‘If everybody were punished for any kind of hate speech*, *I mean especially towards gay persons*, *if they were fined one thousand convertible marks [*national currency, author’s comment] *they would never ever do that again!’*

After considering these statements, it was obvious that the respondents did not feel safe outside their nearest environment, and they were thus mostly isolated in closed environments, which comprised living with their families, less frequently living with their partners, socializing with friends and participating in the work of NGOs that advocated for LGBT rights. One of the respondents claimed that he had to change his place of residence several times after participating in a Pride Parade because he was worried about his safety (J., 27). The analysis of the transcripts found that the participants faced numerous restrictions and that they were seeking refuge because their basic human rights were threatened, starting with the freedom of movement.

*‘Currently I am living alone on the premises of the Association* [NGO that advocates for LGBT rights, author’s comment] *because the situation is not safe and good*. *I can’t really move around so much…(J*., *27)*

‘Here, everybody is afraid. For example, they are afraid of being harassed or they won’t be greeted in the city, so that the others realize what they are…’ (D., 18,5)

‘I feel safe in my small flat, everything outside is insecure’ (N., 38)

Most of our respondents were unemployed or did not have a permanent job, and they often stated that their work for the NGO was professional, although it was in fact voluntary work without any payment. None of the respondents reported having any concrete problems with their employers, but in most cases, they hide their sexual identity from their employer or colleagues (unless they worked for an NGO). Over time, they experienced increased dissatisfaction with their position:

‘I worked in a private company and adapted to it…I took off my earring, made sure I didn’t wear tight clothes, became uniformed, talked about the things I didn’t want to talk about… A man can endure living like this for a year or two, while he can pretend, then he runs away from that place.’

The respondents thought that it was most important to separate their professional and private areas of life and that employees’ lives should therefore not be an issue of interest, neither for employers nor for colleagues. At the same time, they expressed fear that disclosing such information might hinder their future professional development.

‘I adore the profession I have chosen and which I am being educated for and somehow I think it is my way out of all this. I am devoted to social pedagogy and the prevention of the behavioral disorders in children and young people. And suddenly it occurs to me… I am like this and I want to work with children. How will it look? How will some people accept it? Who will let a gay person work with children? And then I realize–I don’t have to go round and keep saying I am gay, I am gay. That’s my privacy!’ (Z., 24)

They also reported numerous restrictions in everyday life, such as problems with renting a flat with a partner or going to a café or other public places with a partner. They longed for very simple things that were considered normal for other people but were forbidden from them; above all, this desire was related to expressing any type of intimacy in public, even holding a partner’s hand. By summarizing those types of restrictions, respondent N. (38) stated that the final outcome and culmination was the inability to legalize homosexual relationships in a homosexual relation:

*‘In the end*, *your partner will fall ill*, *and you won’t even be able to visit him*. *Who are you? Where are you? Are you a member of family? You are not his family*. *I watched a very good short film*, *where a guy couldn’t attend his boyfriend’s funeral*, *his parents didn’t let him go even though they knew they were together*. *And he cried*, *he felt awful*, *and if he had had a paper*, *he could have gone to the hospital and attended his funeral’* (N., 38)

A few respondents identified the high stakes of disclosing their real nature and used a false identity to create their own families and lived with women, and this alternate identity was the source of their greatest vulnerability if their real sexual identity was revealed. Restrictions related to virtual associations also existed, regardless of how important online relationshis occasionally were for spreading information and networking. Several respondents stated that they avoided or were afraid of communicating via Facebook. Some respondents already had negative experiences when Facebook had been used to mock them or disclose their orientation (Z., 24, whose father accidentally found out about his sexual orientation on Facebook and A., 24, whose sister used Facebook to inform others about her brother’s sexual orientation).

*‘Those vultures shared my pictures on Facebook*, *shared my own information… and the police didn’t bother to do something’* (J., 27)

*‘I don’t have a Facebook profile in order to avoid creating an additional problem in my life and because of my family…somebody can see my friends who might be a bit more feminized*. *I don’t want to apologize to anybody’* (N., 38)

When talking about the discrimination they faced, most respondents felt that they were undertreated in almost the same way as other deprived social groups, such as disabled people and national minorities in B&H. They believed that it was not a rare phenomenon for the MSM population to experience multiple discrimination if somebody was HIV-positive or used drugs. Their impression was that the intolerance and hostility created during and after the war in B&H towards other national and religious identities had been shifted to MSM in the same amount and intensity as a common attitude of society as a whole, regardless of MSM’s national ethnicity.

Additionally, the unfavorable economic situation in B&H, as well as in many other transition countries, imposed priorities other than the rights of certain populations such as MSM, who remained marginalized.

‘We are far from parades, we are far from raising the issue of same-sex marriages and I am definitely against these things in environments which haven’t evolved to the point when it can be accepted. We deal with basic existential issues, with providing food, paying bills, surviving… we are still on that level. We still haven’t reached the next, humane level where we can think about ecology and the general welfare and ultimately about human freedoms. This is the step that has yet to be made’ (S., 29)

Nonetheless, the respondents clearly understood that they would remain caught in the ‘vicious circle’ as long as they accepted their current position and until they fought for their rights themselves, until the existing laws were enforced and until those who were devoted to activities and worked in the civil sector clearly expressed their support (for example J., 27 and Z. 24). While discussing what they perceived to be the largest obstacle to achieving basic MSM population rights in a society, respondent Z. (24) specifically noted framing issues related to MSM rights in the wider context of human rights, believing that to be the only real chance of promoting the status of the MSM population:

‘What might be the mistake with the LGBT population is that it demands some particular, additional rights for themselves. My opinion is that we just need to demand that all the rights are respected, i.e., to be equal with others. What I mean is, nothing additional, nothing special… because in that respect what we do is the self-segregation of ourselves!’ (Z., 24)

Different parts of the transcripts also discussed the role of religious organizations in the increased stigmatization of the LGBT population, regardless of the fact that all religions declare understanding, tolerance and equality among people.

‘When I read religious books as a child, either the Koran or the Bible, the things were very simple while reading them… If God creates in his own image, then he also creates people like us. Yet, the attitude coming from the Islamic Community, the Serbian Orthodox Church or the Catholic Church is so terrible and it has such a great power that it is horrible!’ (J., 27)

The respondents were of the opinion that although religious institutions strongly supported totally different nationalistic political forces and did not talk about or prosecute offenses committed by their members (pedophilia, rape), they simultaneously directed intensive condemnation and hate towards members of the LGBT population.

Considering the strong and ubiquitous social discrimination, some respondents talked about their desire (smaller or concrete plans) to continue their lives in a developed Western country, where they believed that the LGBT population as a whole had the same rights as other citizens.

### Healthcare services and MSM

When analyzing the interactions between healthcare services and the MSM population, we identifed the following sub-categories: characteristics of the healthcare service related to the MSM population, relationships and communication with family physicians (GPs), and specific needs and the use of health care services.

Our respondents’ perceptions of accessible healthcare services were not positive; the experiences were diverse, but they most often discussed a ‘stiff’ and outdated system, that could not meet their specific needs. According to them, the greatest barrier to achieving better quality healthcare services for the MSM population was the insufficiently educated doctors, who had negative attitudes towards the MSM population and often breached professional and ethical codes. There were some rare statements from participants talking favorably about their experiences with healthcare professionals. They referred to individuals who were adequately trained to work with people of different sexual orientations or those who were part of teams from NGOs that worked with the LGBT population:

‘The doctor is really kind, really normal, she is well educated, sensible to such things. She went through some training for the MSM population. She has always been a support to me, she behaves like my mother’ (A., 24)

Although our respondents mostly tried to be as invisible as possible in a system where they were not welcome, they were often labeled and stigmatized. One young HIV-positive man (not a respondent), had his serostatus written in large letters on the front page of his medical chart where everybody could see it, including other patients as well as medical professionals. In addition, there were examples of homophobia expressed by some doctors and people of homosexual orientation being banned from donating blood. When participants were characterized as a person with a “sexual orientation disturbance”, this presented additional grounds for stigmatization:

*‘I study social sciences I am well informed about mental health*, *I know some codes*, *according to the ICD 10* [International Classification of diseases version 10, author’s comment], *and then I got a shock*, *I was marked as an ill person*, *as if a sexual orientation disturbance is an illness*. *Then I was prescribed some antidepressives and I have been taking them for eight months without knowing how and what disease they help…’ (Z*., *24)*

‘I think our doctors don’t know much about this issue, they think that being MSM is an illness. And the WHO [World Health Organization] proved it isn’t… and I am also afraid they might tell somebody my orientation’ (B., 21)

The most prominent example of the discrimination experienced in healthcare services was reported by respondent A. (24), whose GP, abstained from providing further medical protection and asked him to take his medical documentation to another doctor after finding out about his sexual orientation (not from him) because of her own religious beliefs:

*‘She just told me–I would like to ask you to change your doctor*. *I was aware that the woman was covered* [Muslim women cover their heads, author’s comment] *and that it was not OK according to her reasoning*, *you understand?(*…*) I didn’t feel good*, *but she kindly asked me to do it*. *If she had been more impolite*, *the situation would have been different* (A., 24)

The respondents mostly stated that their GPs were not familiar with their sexual orientation. The differences in their statements were related to their attitude, to what extent it was important to provide GPs with this information. Most of the respondents still believed it was important for GPs to be informed about their patients’ sexual orientation to be able to deliver the highest quality healthcare service:

‘It is simple—in the world of homosexuals there are risks and specific diseases. Both physician’s help and advice are welcome. It is always good if the physician is informed about his patient’s life. The doctor is supposed to give adequate advice, to give adequate therapies, to treat adequately, to be prepared for whatever happens. Simply speaking, there’s a difference between heterosexuals and homosexuals.’ (D., 16)

‘That’s the identity of that person, it is important for the medical chart. Lifestyles are also different and a person can get a wrong diagnosis in case he/she is silent about something.’ (J., 27)

The oldest respondent was against being asked about his sexual orientation, based on a rational argument that if the question of sexuality was not posed to heterosexual persons, it was unnecessary to insist that homosexual persons should disclose their orientation:

‘The family physician doesn’t know it, and I don’t think it is necessary that he should know it. I think that it would be discrimination if physicians had to know mine or anybody else’s sexual orientation. I am sorry that MSM is the subject. I don’t think that healthcare providers should particularly adapt to the MSM population. There is no difference, what is a problem for hetero(sexual), it is also a problem for homo(sexual).’ (D., 45)

The highest proportion of respondents expressed distrust in healthcare providers, claiming that providers were unable to protect their legal rights, above all the right to data confidentiality. This issue was related to the next sub-category because it reflected the relationship and characteristics of communication with a family physicians. Namely, relationships with family physicians could be defined as superficial and unsatisfactory, as the respondents rarely contacted them, even avoided them, and did not consider them important persons of confidants in their lives or persons with whom they would be glad to share secrets. When physicians were given a hint about problems related to the respondents’ sexuality, they stated that it was not within their competence and, as their best solution, referred them to mental health services. There were also some reports of doctors suggesting some other type of ‘cure’—seeing a *hafiz* (which is a Muslim term for a person who knows the Koran by heart, is well-educated in the Islamic religion and is visited by people when they need help or advice), removing a *sihra* (a word for a black magic spell that somebody has cast on a person) or undertaking a magic ritual; the respondents found these suggestions offensive and considered it unbelievable that a medical professional would provide this type of suggestion:

‘My doctor told me that somebody has cast a sihra on me and suggested that I should see a hafiz. I told her that I am a believer and respect my religion but it is unnecessary. These are not sihras, this is not an illness, this is not black magic…these things cannot be related!’ (A., 24)

‘I persuaded my mother to come to the Center for mental health. She was assigned another doctor. She came home with an idea i.e,. information–a lot of people can get out of that circle when they practice religion i.e,. the devil can be driven out of that person. What is more sad, those were the words of the doctor she saw, a neuropsychiatrist. Later I inquired a bit because that lady is a religious person. I suppose my mother feels bitter, sad, disappointed. She went to the doctor who knows everything. A doctor is an important person in society, a very important segment. And when the doctor gives such information, then I tell her in vain that it is not an illness, that people lead their lives normally, but when she hears something like that…’ (Z., 24).

Lack of trust that their sexual orientation would stay medically confidential was the predominant reason that the respondents were unwilling to provide their family physician with that information, even in the cases when they believed that it was important. They most often had doubts about the professionalism of healthcare professionals, who were supposed to maintain the confientality of data, but in practice, the situation was entirely different. If information related to their sexual orientation was disclosed by healthcare professionals, the respondents perceived that it would mean facing unforeseeable consequences:

‘They don’t deserve to be believed. They neither respect the ethics nor the oaths they made before they became doctors. It would be perfect if doctors stuck to the Hippocratic oath. However, my mother is a healthcare worker and so is my sister, and I know that whatever happens, the information will in some way reach some people’ (K., 21)

‘Usually I don’t go to the doctor, and I am afraid that he might say something to my family. I am scared, I don’t want them to find out from somebody else’ (B., 21)

‘I must not see my doctor at all because knowing my doctor… everybody would know. I really don’t want my family to get that information. When it comes to telling them, I want to be the one who will do that.’ (S., 21)

Therefore, the respondents most often decided to contact healthcare providers only when necessary, to maintain a formal relationship with their family physician and to be cautious to prevent possible compromising situations, as vividly explained by a respondent (S., 29) *‘because of those situations*, *we always have to be five steps ahead!’*

Suspicion related to confidentiality in the doctor-patient relationship and the lack of confidence in their physicians’ competence to meet specific needs were the most represented reasons for the poor use of family physician services, apart from the rare episodes of acute health problems. For health services, our respondents mostly sought and used a psychologist’s help, believing that they were most likely to receive the help they needed from a psychologist. They talked in detail about the support available at centers for psychological support, very emotionally on several occasions, regardless of whether the centers were part of the healthcare system or certain civil sector organizations. These centers were the strongest link in the chain of support provided to MSM and practically the only place where they completely opened up and sought and received help:

‘We need more support, i.e., a psychological support centers, SOS centers. Not only for MSM, i.e., the gay population, but also for all young people who are in a crisis situation or going through critical phases in their lives, whatever; they could contact (centers) by phone or go directly to the psychological support center.’ (Z., 24)

During a time when information is exchanged quickly, it was not surprising that our respondents named a number of comparative needs that indicated that they were informed about the different types of support available and provided to MSM in developed countries abroad: accordingly, they expressed wishes to have those types of opportunities in their own country.

## Discussion

This study presents the first explorative and qualitative study to focus on MSM's perceptions of their own position in modern B&H society, with a particular emphasis on the role of healthcare providers. In this study, we identified very unfavourable findings that confirmed that members of the MSM population are exposed to stigma and discrimination in B&H. Fear, distrust and uncertainty were revealing phenomena presented by all our respondents that further encouraged their conscious self-isolation and concealment of their sexual orientation. The widespread social discrimination among this population has also been documented in preliminary and unpublished results of quantitative research conducted in the same environment, where approximately two-thirds of repondents (67.3%) reported being exposed to public attitudes perpetuating the belief that homosexuality is not normal; additionally, every second respondent was exposed to psychological abuse, mockery and other types of discrimination (author's employer' internal document that is not yet publicly available). That analysis indicated that the MSM population in B&H has generally not disclosed their sexual orientation, and in two-thirds of cases, respondents' family members were not familiar with their sexual orientation; these results imply that our respondents were forced to hide their orientation from their family and constantly pretended to be someone else, which presents a substantial psychological burden.

These findings are in accordance with other studies performed among MSM populations worldwide, especially in developing countries, such as countries in Sub-Saharan Africa, where the rights of MSM are not protected by their states [2]. Although B&H is in the Southeastern Europe region and has a substantially different context from those in Africa, primarily considering the decriminalization of different sexual orientations, the respondents’ sexual orientation was still highly unacceptable socially [25].

Our respondents believed that they would only be able to attain a substantial change in their social civil status if they changed their environment and moved to a developed Western European country, where the status of the homosexual population was perceived to be significantly better. This finding indicates that the motivation for migration is not only economic in nature but is also complimented by the need for a better attitude towards MSM in an environment. This result is in accordance with research conducted by Mole et al. in London among migrants from Central and Eastern Europe [26]. That study also showed that these migrations are often followed by increased sexual activity, especially in the initial period after arrival [26]. These sexual intercourse events are often unprotected, and thus the chances of becoming infected with HIV are much higher, which is similar to the results obtained in other environments characterized by the migration of MSM and a high proportion of MSM among newly HIV-infected people, for example, in San Francisco [27].

The healthcare system is part of the general community and, to a great extent reflects the attitudes of society and the values system. Our findings showed that in rare cases, there were situations in which the respondents were extremely satisfied by their physicians’ attitudes towards them. However, in most cases, our findings indicated that healthcare providers in B&H largely mimicked the behavior patterns typical of all society members regarding MSM, despite the fact that they are supposed to protect human rights and the right to equal treatment. Accordingly, providers’ capacity to provide services needed by this population was hindered because members of the MSM population avoided contacting them, which directly and harmfully affected the health of MSM and perpetuated the vicious cycle of health risks. Specifically, our findings indicated that respondents’ use of healthcare services remained designated to only curative healthcare protection, and even then, they reluctantly asked for help. In addition, the presence of “*medical gossip*” is not uncommon in traditional societies such as B&H [28] and is related to an individual’s fear that confidential information revealed to a healthcare worker might extend beyond their professional rapport and the doctor’s intervention. This fear of gossip has an exceptionally harmful effect on the safety of MSM and their confidence in healthcare providers [28].

Issues related to the rights, needs and attitudes of society regarding population groups of different sexual orientations such as MSM were not prioritized in political agendas nor by researchers in the highly complex historical and geo-political context of B&H from the end of the 20^th^ century and the beginning of the 21^st^ century, and that trend is still present today. Applying Maslow’s hierarchy of needs, post-conflict societies first have to achieve safety, which results from satisfying basic needs; these were all unattainable during the war in the 1990 and the period immediately thereafter. Issues of peace and territorial delimitation, establishment of a political system, and new organization of the destroyed systems and economy created the foundation for newly created social relationships. These issues also refer to the list of priorities that public health authorities were supposed to address; however, the issues and rights of MSM remained marginalized, waiting for more prosperous times and conditions. A report issued by the United Nations Developed Program (UNDP) described similar findings in other surrounding countries (Serbia, Croatia and Montenegro) that also had complex (and very similar) social, cultural and political circumstances in the recent past [29].

### Limitations of the study

This study has certain limitations worth mentioning. One limitation is related to the accessibility and selection of the respondents, who were recruited through the websites of activist organizations in B&H and who gravitated towards the major cities in B&H where these organizations operate. Recruitment via the Internet certainly excluded MSM who were not familiar with these NGOs or who were not regular Internet users for whatever reason. Our sample was thus certainly biased; however, this method was the most feasible option for studying stigma and discrimination among MSM in B&H.

In addition, a similar proportion to B&H EMIS sample had tertiary education, suggesting that these two studies were accessing a similar MSM population (7).

We believe that potential respondents, who had disclosed or were latent members of the MSM population and could not be reached because they were not in the network of the above-mentioned organizations, could provide even more drastic answers related to their experiences in the community and with healthcare services. We presume that the estimated size of the MSM population, at 0.57% of the total male population in B&H, does not present the real size of this population group [13] and underestimates the true prevalence.

The results of our study provide important knowledge for creating policies, activities and interventions, particularly in the healthcare services, which would benefit significantly from education of healthcare professionals about the needs and rights of MSM. There is numerous evidence in the scientific literature that such trainings, whether they are conducted in the form of ‘live’ seminars or via online education, can significantly contribute to changes in the relationships of healthcare professionals with MSM, can increase the respect for their basic human rights and can decrease the degree of discrimination that they experience [30–32]. In the long term, such strategic investment in human resources in the healthcare system might affect the existing inequalities in health care, enabling better quality preventive programs for MSM. Adequate and timely reactions to the identified risks that MSM are exposed to could decrease the extent of HIV infection, which unfortunately has witnessed a growing trend in many countries worldwide [33].

Healthcare protection of MSM in B&H cannot be achieved as long as stigma and discrimination are widely spread among healthcare professionals and society in general. Further intervention studies are needed to identify the most successful practices that could assure respect for diversities and equal health for all.

## Conclusion

MSM in B&H experienced various levels of stigma, discrimination, prejudices and inequities in different segments of society as well as in the health care sector, which prevented them from fully achieving their human and health potential. There are numerous opportunities to implement educational trainings and structural reform to create a society that provides and guarantees equal opportunities for all.

## Supporting information

S1 File Interview guide in English and Bosnian language. (ZIP)
