# Supplementary material for: ‘The Devil has entered you’: A qualitative study of Men Who Have Sex With Men (MSM) and the stigma and discrimination they experience from healthcare professionals and the general community in Bosnia and Herzegovina
Source: PLoS One. 2017 Jun 7;12(6):e0179101. doi: 10.1371/journal.pone.0179101 (PMC5462409; doi:10.1371/journal.pone.0179101)
Supplement: S1 File — (ZIP) [file pone.0179101.s001.zip › S1_File/S1B_File. Interview Guide in Bosnian Language.docx]

VODIČ ZA INTERVJUISANJE

Poštovani,

Ja sam _______________________ i željela bih da danas razgovaramo o temama koju su značajne za unapređenje zdravlja MSM u BiH i posebno značajne za prevenciju HIV-a i drugih seksualno prenosivih infekcija. Današnji intervju dio je istraživanja prevalence HIV-a, Hepatitis B, Hepatitisa C i Sifilisa u populaciji MSM u BiH koje se provodi kao dio projekta „Povećanje univerzalnog pristupa za populacije pod povećanim rizikom u BiH“. Vaše učešće u ovom istraživanju je potpuno dobrovoljno i anonimno. Anonimnost u istraživanju znači da se nigdje ne pominju lični podaci ispitanika. Ja vas neću ni pitati za vaše lične podatke, osim imena, radi lakše komunikacije, i uvjeravam vas da vaši odgovori ni na koji način neće biti u vezi sa njima.

Neka pitanja su lične prirode i odnose se na individualna iskustva, mišljenja i zapažanja, a vaši odgovori će nam pomoći da bolje sagledamo potrebe pojedinih populacionih grupa i prepreke u njihovoj realizaciji, kako bi se poboljšala dostupnost odgovarajuće zdravstvene zaštite. Vi u svakom trenutku možete da prekinete intervju ili da ne odgovorite na neko pitanje.

Prije nego razgovaramo o vašoj saglasnosti za učešće u istraživanju, voljela bih da znam da li vas još nešto interesuje, da li vi imate neko pitanje za mene.Ukoliko nemate (više) pitanja ću da pitam vas „Da li ste saglasni da učestvujete u istraživanju“?

Radi poštovanja etičkih principa i protokola istraživanja moram da vas zamolim za informisani pristanak. Informisani pristanak znači da ste upoznati sa svrhom istraživanja i da dobrovoljno želite da učestvujete u istom. Ukoliko ste saglasni sa učešćem molim vas da ovaj formular potpišete na način koji vama najviše odgovara (šifra, skraćeni potpis, potpis ili nešto slično).

Informisani pristanak DA _________________________ NE

Zbog lakše analize podataka, potrebno je da se intervju zvučno snimi, što će biti dostupno samo članovima najužeg istraživačkog tima. Da li ste saglasni da se intervju snima?

**DA NE**

**Intervjuer: ___________________________________**

**Datum: ____________**

**Vrijeme početka intervjua: _________________**

**Vrijeme završetka intervjua: _________________**

**Lista pitanja za razgovor**

|  | **Ključna pitanja** | **Dodatna pitanja** | **Zabilješke** |
| --- | --- | --- | --- |
| **1.** | Koliko imate godina?  Da li ste završili školu i koju?  Da li ste zaposleni? |  |  |
| **2.** | S kim živite? Da li ste u braku ili nekoj drugoj vrsti zajednice? | Koji je glavni razlog za život u zajednici? |  |
| **3.** | Da li su po vašem mišljenju, u sredini u kojoj živite, MSM prihvaćeni kao i osobe heteroseksualnog opredjeljenja? | Ako nisu, zašto mislite da je to tako? Možete li mi to detaljnije pojasniti ili navesti neki primjer? |  |
| **4.** | Kako biste definisali vaše seksualno opredeljenje? Koje je vaše seksualno opredjeljenje? | Možete li mi molim vas pojasniti šta to tačno znači? |  |
| **5.** | Šta članovi vaše porodice misle o MSM? | Kako su saznali? Da li ste im rekli ili su saznali na neki drugi način? |  |
| **6.** | Da li vaša porodica zna za vaše seksualno opredjeljenje? Kako je porodica primila informaciju o vašem seksualnom opredjeljenju? | Da li su podržali vaše opredjeljenje, da li podržavaju vašu seksualnost? Da li je uslijedila promjena ponašanja prema vama, ako jeste o kakvoj promjeni se radi? |  |
| **7.** | Šta članovi vaše porodice misle o tome? Šta je sa predrasudama prema drugim pripadnicima MSM populacije? | Ako da, zašto mislite da je to tako? Na koji način su predrasude ispoljene? |  |
| **8.** | Šta vaši prijatelji misle o MSM? Da li vaši prijatelji znaju za vaše seksualno opredjeljenje? | Da li je to uticalo na vaše prijateljstvo? Na koji način? Da li su neka prijateljstva bila narušena? Kako ste se tada osjećali? |  |
| **9.** | Ukoliko ste zaposleni, da li vaš poslodavac zna za vaše seksualno opredjeljenje? | Ako da, da li je to poslodavac znao prije zapošljavanja ili je saznao kasnije? Da li se po vašem mišljenju ponašanje poslodavca prema vama razlikuje od ponašanja prema drugim zaposlenim? Ako da, zašto misliš da je tako? Da li na poslu doživljavaš neugodnosti zbog svog seksualnog opredjeljenja (kakve, od koga, koliko često) |  |
| **10.** | Da li vaš porodični doktor zna za vaše seksualno opredjeljenje? | Da li ste ikada razgovarali o vašoj seksualnosti? Kakav je stav vaš porodični doktor ima prema vašoj seksualnoj orijentaciji? Da li vas je porodični doktor bilo kada izložio nekoj vrsti neugodnosti (odbio uslugu, nepotrebno uputio drugom doktoru i sl.)? Ako da, šta je po vašem mišljenju glavni razlog za takvo ponašanje? |  |
| **11.** | Kakva se osjećate u vezi sa vašom seksualnošću? | Da li možete otvoreno govoriti o svojoj seksualnosti (i sa kim)? Koliko često otvoreno govorite o svojoj seksualnosti? Da li je tokom vremena bilo promjene osjećanja? Šta mislite zašto je to tako? |  |
| **12.** | Da li ste ikada osjećali da vam je potrebna stručna pomoć? | Zašto mislite da je to tako?  Ako da, da li ste potražili stručnu pomoć i od koga? |  |
| **13.** | Po vašem mišljenju da li su MSM više ili manje izloženi riziku od oboljevanja od nekih bolesti? Kako procenjujete rizik od oboljevanja od HIV-a i drugih STI od ostatka populacije? | Šta mislite, zašto je to tako? |  |
| **14.** | Po vašem mišljenju, šta bi trebalo da se uradi kako bi se poboljšalo sprečavanje prenosa HIVa i polno prenosivih infekcija? | Možete li mi to bolje pojasniti? Možete li mi navesti neki primjer? |  |
| **15.** | Šta je po vašem mišljenju najveća prepreka u poboljšanju zdravstvene zaštite? | Na koji način mislite da zdravstvena zaštite može da se poboljša, posebno kada govorimo o MSM? Šta nedostaje? |  |
| **16.** | Da li možda želite da dodatne nešto što mislite da je značajno za ovaj intervju, odnosno što bi zajednica trebala znati? |  |  |
