# Supplementary material for: ‘The Devil has entered you’: A qualitative study of Men Who Have Sex With Men (MSM) and the stigma and discrimination they experience from healthcare professionals and the general community in Bosnia and Herzegovina
Source: PLoS One. 2017 Jun 7;12(6):e0179101. doi: 10.1371/journal.pone.0179101 (PMC5462409; doi:10.1371/journal.pone.0179101)
Supplement: S1 File — (ZIP) [file pone.0179101.s001.zip › S1_File/S1A_File. Interview Guide in English Language.doc]

INTERVIEW GUIDE

My name is ______________________ and today I would like to talk to you about the issues which are relevant for promoting the health of MSM in B&H and which are particularly important for the prevention of HIV and other sexually transmitted infections. Today's interview is a part of the study related to HIV prevalence, Hepatitis B, Hepatitis C and syphilis among MSM population in B&H and which is being carried out as a part of the project 'Increase of the universal approach to populations at higher risk in B&H'. Your participation in this study is absolutely voluntary and anonymous. The term anonymous in the study means that personal information about the interviewees is not revealed anywhere. I am not going to ask you about your personal information, apart from your name, which is necessary for easier communication and I can assure you that your responses will by no means be related your personal information.

Some questions are personal and they relate to individual experience, opinions and observations, and your responses will help us to get a better insight into the needs of certain population groups and the obstacles in realizing them, with the aim to improve the availability of the appropriate health care. You are entitled to stop the interview at any time or you can refuse to give an answer to a certain question.

Before we go on to talk about your consent for participating in the study, I would like to know if there is something else that you are interested in or whether you have some questions to ask me. If you do not have any (any more) questions, I am going to ask you this question ' Do you agree to participate in this study'?

Following the ethical principles and the study protocol I have to ask for your informed consent. Informed consent means that you are informed about the aim of the study and that you want to take part in it voluntarily. If you agree to participate, I would like to ask you to sign this form in the way which you find most appropriate (code or short signature or initials or full signature or something similar).

Informed consent	YES_________________________ NO

In order to enable easier analysis of the data it is necessary to make the voice recordings of the interview, to which only the key members of the research team will have access. Do you give your consent that the interview can be recorder?

**YES	NO**

**Interviewer: ____________________________________**

**Date: ________________________________**

**The interview started at: _____________________________**

**The interview ended at: ____________________________**

**List of interview questions**

|  | **Key questions** | **Additional questions** | **Notes** |
| --- | --- | --- | --- |
| **1.** | How old are you?  Did you finish school and what school did you finish?  Are you employed? |  |  |
| **2.** | Who do you live with? Are you married or you live in another type of relationship? | What is the main reason for living in that relationship? |  |
| **3.** | In your opinion, are the members of MSM accepted in the environment where you live in the same way as the persons of heterosexual orientation? | If not, why do you think this is so? Can you explain that in detail or give some examples? |  |
| **4.** | How would you define your sexual orientation? What is your sexual orientation? | Can you please explain what it means exactly? |  |
| **5.** | What do the members of your family think of MSM? | How did they find out? Did you tell them or they found out in some other way? |  |
| **6.** | Is your family aware of your sexual orientation? How did your family accept the information about your sexual orientation? | Did they support your orientation; do they support your sexuality? Did any change in behavior towards you occur, if yes, what kind of change is it? |  |
| **7.** | What do the members of your family think of it? What about the prejudices towards other members of MSM population? | If yes, why do you think this is so? In what way are those prejudices expressed? |  |
| **8.** | What do your friends think of MSM? Are your friends aware of your sexual orientation? | Did it have any influence on your friendship? Did your relationship with friends suffer? How did you feel then? |  |
| **9.** | If you are employed, is your employer aware of your sexual orientation? | If yes, was your employer aware of it before you were employed or he found it out later? In your opinion, does the behavior of your employer towards you differ from the behavior towards other employers? If yes, why do you think this is so? Do you experience any kind of discrimination at work due to your sexual orientation (what kind, by whom, how often) |  |
| **10.** | Is your family doctor familiar with your sexual orientation? | Have you ever talked about your sexuality? What is the attitude of your family doctor towards your sexual orientation? Did your family doctor ever put you in an embarrassing situation (refused to provide treatment, unnecessarily referred you to another doctor etc.)? If yes, what is in your opinion the main reason for such behavior? |  |
| **11.** | How do you feel about your sexuality? | Can you openly talk about your sexuality (and to whom)? How often do you openly talk about your sexuality? Have your feelings changed over time? Why do you think this is so? |  |
| **12.** | Have you ever felt that you need professional help? | Why do you think this is so?  If yes, did you look for professional help and who from? |  |
| **13.** | In your opinion, are MSM more or less exposed to the risk of contracting some diseases? How do you estimate the risk of contracting HIV or other STIs compared to the rest of the population? | Why do you think this is so? |  |
| **14.** | In your opinion, what should be done to improve the prevention of the transmission of HIV and other sexually transmitted diseases? | Can you clarify that a little? Can you give me an example? |  |
| **15.** | What is in your opinion the biggest obstacle to the improvement of health care? | In your opinion, how can heath care be improved, especially when talking about MSM? What is lacking in health care? |  |
| **16.** | Do you want to add something to this interview that you find important, i.e. something that the community should know? |  |  |
